# Prophylactic Treatment of Abortion

**Published:** 1886-10-20

**Authors:** J. H. Coulter

**Affiliations:** Peoria, Ill.


### PROPHYLACTIC TREATMENT OF ABORTION.

BY J. H. COULTER, A. M., M. D., PEORIA, ILL.

The treatment of abortion in its most important phase must necessarily be prophylactic. Abortion may occur from some morbid condition of the uterus, from a constitutional disease or from a predisposition, as we say "habitual abortion." There may be other causes, but they are either unusual or irrelevant to the present notice, hence I leave them unmentioned.

So far as I can learn the use of chlorate of potash in this condition, as a prophylactic medicament, was first proposed by Dr. Mitchell, an English obstetrician. The French and Americans used other remedies, claiming for them equal efficacy; among them were viburnum prunifolium, mercury, opium, tampons, etc. Of these, the first has fallen into disuse, and its efficiency questioned on all sides. Mercury was used in those cases particularly where the abortion was due to syphilis, and in such cases its usefulness cannot be questioned. Prof. Kleimachte says, "the internal administration of all such remedies as opium is absolutely without advantage."

The exact physiological action of chlorate of potash is a question of discussion. The potash salts are to a limited extent depressant in their action on the brain and nervous systems. But the most noticeable action of the chlorate of potash is, it tends to decrease the excretions of glands and mucous membranes, and to this action, to some extent, is its usefulness in the above cases attributable; certainly, though, it has in its sedative action its most impoportant sphere. Prof. Von Meering, of Straasburg, made a very close and thorough examination and study of the action of this drug. See *Peoria Medical Monthly*, Vol. VI, p. 635.

But it was not my purpose so much to discuss the action of the drug as to notice its practical value in the cases above referred to, and in this connection allow me to cite the history of two or three cases which recently came in my experience.

### CASE I.

Mrs. S——, aged thirty; of anæmic and weak constitution; married four years. About two and a half years ago, when under treatment for a pelvic neuralgia, being then near the end of the third month of gestation, the attending physician applied a strong current of electricity to the uterus and abdomen. The result was the induction of an abortion which took place in twelve hours. She made a slow but fair recovery, and in the course of one year again became pregnant. I may add she was, after the abortion until this time, subject to dysmenorrhœa, but was one of the few who become pregnant under such circumstances. She progressed quite normally so far as known until the fourth month, when the symptoms of abortion appeared. A physician was called but failed to prevent an abortion, which took place forty-eight hours after the first symptoms of abortion appeared. This time the recovery was much more rapid and apparently complete; no further dysmenorrhœa for sometime. Such was the history of the case as given me when I was called one year ago to advise concerning, and prescribe for, the supposed sterility. An examination showed considerable degree of anteversion. This, however, yielded to treatment very satisfactorily, and in a few months pregnancy ensued. Being aware of the previous abortions, as well as the fear of another, I began about the second month to administer small doses of potassium chlorate once daily. But again about the same period of gestation slight symptoms of abortion appeared, though the flow was slight, and the other symptoms scarcely more marked. I had already gradually increased the dose, and was now giving 12 grains three times daily. With this the unfavorable symptoms soon disappeared, and I recently attended her at the birth of a healthy, full term male child, weighing nine pounds. I consider the second abortion due in a great measure to her nervous and solicitious mental condition, rather than to any natural or predisposing cause entirely; and I feel as confident that without the drug or its equivalent, abortion in the last gestation would have been inevitable.

### CASE II.

Mrs. K——, German; forty-three years old; married twenty-two years; oldest child 20; five children living, all healthy but one, who was scarcely an eight months child; has aborted at least five times.

On April 2 at 11 p. m., I was called to see the case, which presented, with the above, the following conditions: Patient very much weakened and discouraged; being in the seventh month of gestation, and also certain an abortion was inevitable; the flow had been constant for three days, though not alarming in quantity until the present evening, when, as she was retiring it suddenly assumed serious proportions, and labor pains at once came on. The os uteri did not show the least dilatation, and I saw that the pains were to a great extent "false pains." A hypodermic injection of morphine, $\frac{1}{4}$ gr., soon relieved all except the excessive hemorrhage; for this I used cold water injections freely, and in ten minutes all was quiet. I ordered absolute rest for several days and left the following:

℞. Potassium chlorate............................6 ʒ,

Syr. lemonis..................................$1\frac{1}{2}$ ℥,

Aq. dist., q. s. ad................................3 ℥.

M. Sig—A teaspoonful every three hours, gradually decreasing the dose to a half teaspoonful twice daily.

I however did not entirely discontinue its use. In three days the patient was up walking around the house, eating and sleeping well, until May 27, when I delivered her of a fully developed female child. The child was not nearly so healthy as that in Case I, although the mother seemed much stronger and in every way the healthier of the two. I do not think, however, that such doses of chlorate of potassium would affect the child so unfavorably. I should much rather attribute its condition to the threatened abortion *per se*.

In cases where syphillis is known or even suspected I would not advise dropping the mercury, but rather alternate the two by shorter or longer periods as the case may require.

Except in urgent cases always give the drug after meals, as its deleterious effects seem to be manifested only when the salt is absorbed by an empty stomach, and when for some reason its elimination is incomplete or insufficient.

The drug commends itself to the practitioner for many reasons. Its ease of administration, its safety and its efficacy are not the least worthy of notice. It is a tried friend of every practitioner who has given it a fair trial. We see our oldest and most successful practitioners sticking so close to some such old drugs, that we want to say "old fogy" and substitute some recent "proprietary" for it. And if, perchance, we do get an opportunity, then our "elegant preparation" fails to reach our anticipations in nine times out of ten.

I am not the one to berate or discourage in any way the advance in any direction of our noble profession; but just now may it not be possible we young Americans are fast approaching an extreme opposite to this "fogyism" we so hate to come in contact with? We should be certain we are soaring on wings which cannot be melted by the combined suns of all other "pathies."—*Peoria Medical Monthly*.

